# Tackling health professionals’ strikes: an essential part of health system strengthening in Kenya

**DOI:** 10.1136/bmjgh-2018-001136

**Published:** 2018-11-28

**Authors:** Grace Irimu, Morris Ogero, George Mbevi, Celia Kariuki, David Gathara, Samuel Akech, Edwine Barasa, Benjamin Tsofa, Mike English

**Affiliations:** 1 Department of Paediatrics and Child Health, University of Nairobi, Nairobi, Kenya; 2 Kenya Medical Research Institute (KEMRI), Wellcome Trust Research Programme, Nairobi, Kenya; 3 Department of Paediatrics Mama Lucy Kibaki Hospital, Nairobi City County, Nairobi, Kenya

**Keywords:** strikes, human resource for health, collective bargaining agreement, devolution of health services, constitution

**Summary box**

- Since 2013, Kenya's public health sector has been affected by frequent short strikes, culminating in nationwide strikes lasting a total of 250 days by doctors and nurses in a span of 11 months in 2016/17.
- Health professionals have the right to go on strike, but their strikes crippled health services with almost no public hospital inpatient services being provided, thus violating people’s right to healthcare.
- To avoid similar instances in the future, mechanisms should be established for dispute resolution, anticipating and pre-empting changes within the health system that result to conflict between parties.
- There are no ‘magic bullets’ to avert all problems due to these strikes in what are complex, politically managed and highly professionalised health-sector organisations.
- Reactive solutions such as sacking striking workers, jailing trade union officials neither address the underlying problems nor build resilience of the health system.

## Introduction

Since devolution of healthcare services in 2013, the Kenyan public health sector has been affected by frequent short and often localised strikes.^1^ These were followed by a public-sector nationwide doctors’ strike lasting 100 days (from 5 December 2016 to 14 March 2017) and then the nurses’ strike lasting 150 days (from 5 June to 1 November 2017), a total of 250 strike days in a span of 11 months, referred to hereafter as the 2017 strikes. The strikes resulted from a complex chain of events briefly outlined below.

A new 2010 Kenya Constitution gave every worker (with some exceptions for disciplined armed forces) the rights to join a union, engage in collective bargaining and the freedom to strike, linked to supporting rights to fair remuneration and reasonable working conditions.^2^ This constitution also devolved all primary and secondary health services to 47 new semiautonomous county governments. Initial plans were to progressively transfer functions over a 3-year transition period from 2013.^2^

However, political pressures resulted in devolution of health services occurring over 6 months to counties that had limited capacity in human resource for health (HRH) and medical supplies management. Salary delays and challenges managing career progression agreements and postgraduate training schemes, long stock-outs of essential medicines and discrimination against persons perceived to be outsiders were reported from some counties in the 3 years preceding the 2017 strikes.^1^ During this period, doctors and nurses formed trade unions.^3 4^ The doctors negotiated collective bargaining agreements (CBA) on pay and working conditions, which was reportedly signed by the national government in 2013 but not implemented and county governments did not then accept responsibility for it.^5^ Both the national and county government are reported to have failed to sign the draft nurses’ CBA that encouraged them to return to work after a 2-week strike in December 2016 resulting in the prolonged 2017 nurses’ strike. Table 1 summarises the events that led to the 2017 strikes.

**Table 1:** Occurrence of events that led to the doctors’ and nurses’ strikes in Kenyan public hospitals

Year	Events	What the event entailed	Implications
2010	The promulgation of the 2010 constitution^2^	Kenya adopted a rights-based approach and devolved the responsibility of public health service delivery of primary and secondary health services to 47 counties. Establishment of counties’ structures, capacity building and transition planned to take 5 years.^2^	The constitution (Article 41) gave all types of workers (except disciplined armed forces) the right to: (1) fair remuneration, (2) reasonable working conditions, (3) form, join or participate in the activities and programmes of a trade union and (4) go on strike. Every trade union was given the right to engage in collective bargaining^2^
2011–2012	Doctors’ Union formed^3^	Doctors’ Union called for a strike citing poor working conditions and poor wages. Musyimi Task Force formed (six government officers and six Union members) to address health sector issues raised by the Union.^15^	Strike lasted a few days Musyimi Task Force agreed on Return to Work Formula including formulation of a CBA and a proposal to establish Health Service Commission to manage service delivery.^15^
2013	A CBA drafted by Doctors’ Union and Ministry of Health and reportedly signed by both parties	CBA aimed at aligning remuneration of doctors with the labour market by demanding a 300% pay rise to all medical practitioners and compensation if working>40 hours per week, review of job groups, promotions, deployment and recruitment of more doctors.	The county governments were not signatory to the CBA and they felt negotiation should have been with each county government,^5^ The national government did not submit the CBA to the industrial court for registration.
2013	New government formed following general elections	Devolution of healthcare launched officially. June 2013, all county level functions speedily transferred to the counties in response to demand by the newly elected county governors.	Some counties lacked structures and capacity to take the new roles in management of human resource for health and medical supplies resulting in delayed salary processing and stock out of essential medicines.^1^ A nation-wide health professionals strike in late 2013. The strike called off after several weeks when the striking health professionals were sacked.^1^
2013	Registrar of Trade Unions directed by the court to register nurses’ union^4^	Nurses’ Union formed	The Union negotiated a 25%–40% pay raise, improvement of all its members working conditions and the absorption of all its members into permanent and pensionable schemes.
5 December 2016 to 15 March 2017	Doctors’ nation-wide strike in demand of fulfilment of the 2013 CBA	The strike affected all public hospitals under national and county governments (including the tertiary and university teaching hospitals).	Several attempts by the employer and doctors to negotiate made but marked with power games, mistrust, miscommunication, jailing of union officials and political interference.^5^ The 100-day nationwide strike ended after agreement.
5−14 December 2016	Nation-wide nurses’ strike	A duty resumption agreement signed on 14 December 2016 between the national and the county government and nurses’ union to end the strike.^8^	Award of specified allowances agreed on.^8^ Salary issues were to be addressed in a comprehensive CBA.
5 June to 2 November 2017	Nation-wide nurses’ strike	Strike attributed to failure of the national and county governments to sign a CBA on pay and working conditions.^8^	County Governors persuaded the County Nursing Officers to call nurses back to work, a strategy that worked in some counties (affected two Clinical Information Network hospitals), though it was disapproved by the Union officials. 150-day nation-wide nurses’ strike ended after agreement^8^ (CBA was signed later in March 2018)

CBAx, Collective bargaining agreement.

## The consequences of the strikes on hospital admissions

Data from 13 county public hospitals that provide first-referral care illustrate the effects of the 2017 strikes. These hospitals are part of the Clinical Information Network (CIN) since 2013/2014 whose aim is to improve quality and utilisation of hospital data. While they are not a nationally representative sample of hospitals, we believe their data reasonably mirror the pattern of hospital admissions in other county hospitals (they are described in more detail elsewhere).^6^ Data on paediatric admissions were taken from the ongoing CIN surveillance platform and for other wards were obtained from the hospitals’ health-records departments.

We present a 2-year data pooled from all hospitals on the number of admissions per month in the four major inpatient wards from January 2016 to December 2017. We use the data from January 2014 to December 2015 for the paediatrics and maternity wards to demonstrate annual patterns of admissions and any seasonality that might exist.

**Figure 1:**
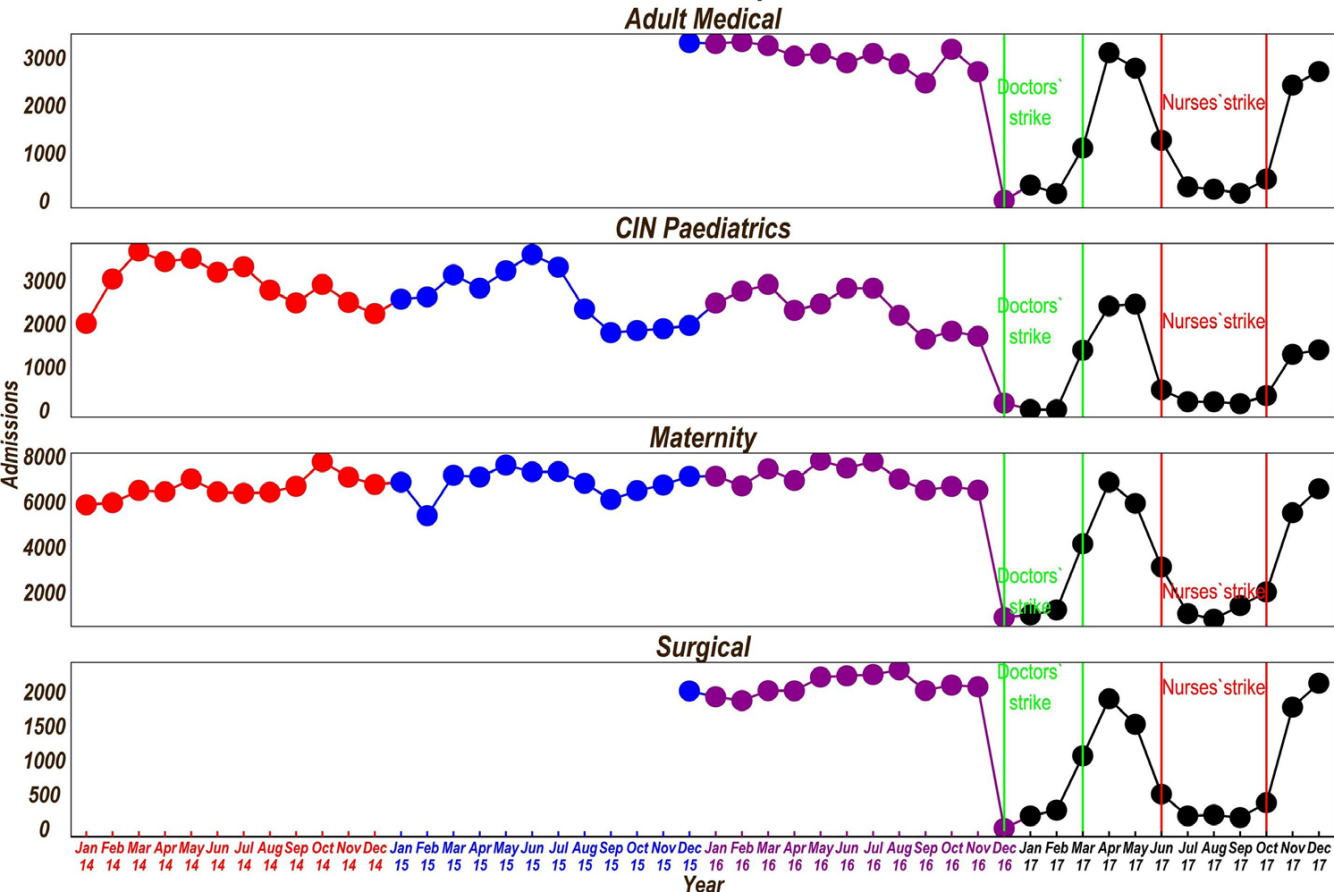
Admissions over time across wards in 13 CIN hospitals. CIN, Clinical Information Network.

During both the doctors’ and nurses’ 2017 strikes, there were marked reductions in admissions in all the four major disciplines—obstetrics, paediatrics, surgical and adult medicine (figure 1). Exploration of hospital-specific data (available on request) demonstrates varied responses to the strikes across hospitals and wards. There was limited continuing admissions in different hospitals in specific wards (maternity (n=1/13), adult medical (n=1/13) and surgical (n=1/13)); resumption of services before the strikes officially ended (in two maternity wards and across all wards in two hospitals) and use of locum nurses to keep all the wards open (one hospital). During the entire 250 days of the strike, four hospitals had almost no admissions at all.

Considering the admissions in the prestrike year (December 2015 to November 2016), we speculate that a total of 183 170 individuals (including that each maternity admission produced one new-born) did not receive admission care in these 13 hospitals during strike year (December 2016 to November 2017). This included 59 965 maternity patients (and the same number of newborns), 24 762 medical patients, 20 309 paediatrics and 18 169 surgical patients. There are 65 similar level referral hospitals in Kenya (Kenya Master Facility List), and we tracked data from 13 of these that were part of CIN, suggesting that preventable deaths likely occurred on a massive scale. Private and faith-based hospitals reported increased admissions and mortality over this period.^7^ Typically, county hospitals see many more outpatients than inpatients and so the total number of lost episodes of care in the public sector would be considerably higher.

## No ‘Magic bullets’

There are no ‘magic bullets’ to avert all problems due to these strikes in what are complex, politically managed and highly professionalised health-sector organisations. Where there is a right to health professionals’ industrial strike, appropriate mechanisms are required to avert harms. Reactive solutions such as sacking striking workers and jailing trade union officials neither address the underlying problems nor build resilience of the health system.^1 5 8^ Instead, there is a clear imperative to improve the management of HRH and develop proactive policies and risk management plans that nurture systems’ ability to withstand crises while maintaining functions.^9^ Data from the 13 hospitals in the CIN indicate that two of the hospitals had 1-day strikes in 2014, seven had strikes ranging from 1 to 49 days in 2015, and in 2016 (January to November), all hospitals had strikes for periods ranging from 27 to 59 days. Such data suggest there were clear warning signs of deteriorating relationships that did not prompt mitigating actions.

HRH strikes raise a moral dilemma with the potential of causing harm to patients, violating professional ethics and the Hippocratic oath. Yet, HRH strikes may be justifiable if there is evidence of long-term benefits to the doctors, patients and overall quality of care.^10^ Importantly, in Kenya, although the public sector is said to provide 48% of all Kenya’s healthcare, it provides a much greater proportion of inpatient hospital care particularly for the 36.1% (18 million) of Kenya’s population living in poverty (Kenya Economic Survey 2018). Strikes thus disproportionately affect the poor who are unable to afford private sector alternatives. This population therefore deserves special ethical consideration when health professionals’ strikes are contemplated.

## Rebuilding relationship among stakeholders

National and county healthcare leadership need to rebuild relationships with professional groups who, in turn, need to better define their own professional norms and values^11^ so that, during health system level ‘shocks’ professional and societal values might be better aligned. Spaces need to be created for all groups to contribute to planning of healthcare and policies that are cognizant of economic and social realities spanning training, deployment of staff, management of performance and definition of working conditions.^12^ Such efforts are needed to rebuild trust.

Specifically, there needs to be a national and inclusive dialogue (with patient representation) to develop minimum operating requirements in the event of strikes to prevent the near total collapse of the public health system seen in 2017.^9^ This is especially important as the Kenya Constitution permits HRH strikes and also states that ‘A person shall not be denied emergency medical treatment’.^2^ Special consideration therefore needs to be given to handling of emergencies, ensuring continued care of already hospitalised patients and how to safely staff vital facilities, such as the emergency rooms, obstetric and neonatal departments.^13^ In some countries, for example, senior clinicians continue to give services when junior staff are on strike.^14^ This arrangement provides at least some cover while maintaining positive elements of an overarching professional ethos. These risk management plans are best agreed on outside times of crisis and might be enshrined in specific agreements or even in the Constitution or Government legislation. Such actions need to be taken now.

Going further, a specific forum could be created, including senior representatives of all parties, to regularly meet to discuss health system challenges before they escalate to strike action. Such a forum may have facilitated discussions on the issues raised by the Doctors’ Union that were articulated in the 2012 Musyimi Task Force Report.^15^ It could also help in planning how best to deliver healthcare in times of natural or other hazards (epidemics, natural disasters) and should have the opportunity to discuss implementation of major proposed health-system reforms such as devolution/decentralisation of health services. The forum should include representatives from national and county governments, health professional associations and their unions, medical teaching institutions, private/faith-based healthcare providers, communities and partners in healthcare with an aim to build collaboration among stakeholders. As a further precaution, mechanisms for effective, fair and independent arbitration should be developed and agreed to by all parties in advance of any future strike action. Most important is that employers and employees should honour agreements made and act in good faith for the benefit of the whole population and especially its most vulnerable groups.

## Conclusion

The health professionals’ strikes crippled the health services with almost no public hospital inpatient services being provided and violating people’s right to healthcare. To avoid similar instances in the future, the specific roles and responsibilities of the national and county governments should be clearly defined in a devolved health system. Mechanisms should be established for dispute resolution, anticipating and pre-empting changes within the health system that result to conflict between parties. Complexity of health systems should be taken into consideration in problem and solution identification.
